# Overexpression of *PeBBM2* and *PeWUS* Genes via Carbon Nanotube-Based DNA Delivery Enhances the Callus and Shoot Formation in *Phyllostachys edulis*

**DOI:** 10.3390/genes17060598

**Published:** 2026-05-22

**Authors:** Yiqian Ding, Ruotong Xu, Chao Xu, Xiaohong Zhou, Mingbing Zhou

**Affiliations:** 1 College of Horticulture and Forestry, Tarim University, Alar 843300, China; 2 State Key Laboratory for Development and Utilization of Forest Food Resources, Bamboo Industry Institute, Zhejiang A&F University, Hangzhou 311300, China

**Keywords:** *Phyllostachys edulis*, genetic transformation, BABY BOOM (BBM), WUSCHEL (WUS), single-walled carbon nanotubes

## Abstract

**Background/Objectives**: *Phyllostachys edulis* is the most widely distributed and economically important bamboo species in China. However, the genetic transformation in *P. edulis* is still limited by a long regeneration cycle and low regeneration and transformation efficiency. Carbon nanotube-based delivery systems in plants have the advantages of simplicity, rapidity and low cost. Moreover, morphogenetic regulators BBM (BABY BOOM) and WUS (WUSCHEL) play significant roles in plant regeneration. **Methods**: Here, immature zygotic embryos were used to induce *P. edulis* callus, and using single-walled carbon nanotubes (SWNTs)-based delivery technology, *PeBBM2*, *PeWUS*-DNA (with introns) and *PeWUS*-cDNA (without introns) were introduced to *P. edulis* callus either individually or in combination. **Conclusions**: The results showed that the 0.9–1.0 mm (long axis) embryos exhibited the lowest contamination rate and the highest induction efficiency. Moreover, the results indicated that the co-transformation of *PeBBM2*-*PeWUS* more effectively boosted the growth area of the callus. However, only the *PeBBM2*-overexpression callus could form shoots. Compared with the wild type, the *PeBBM2*-overexpression lines showed reduced expression of *AGL15* and increased expression of *IAA30* and *YUC*. In conclusion, these findings suggested that SWNTs-mediated DNA delivery is a potential strategy for the genetic transformation of *P. edulis* callus. Additionally, the findings indicate that the *PeBBM2* and *PeWUS* genes can accelerate callus enlargement in *P. edulis*, whereas *PeBBM2* might play a more important role in shoot formation. This study provides a basis for developing a genetic transformation system for plants based on SWNTs-mediated DNA delivery and morphogenetic regulators.

## 1. Introduction

Plant DNA delivery methods mainly include agrobacterium-mediated, biolistic gene, and carbon nanotubes (CNTs)-based delivery [1,2]. Among these, agrobacterium-mediated transformation is widely used for the efficient and stable transfer of large DNA constructs. However, its application remains limited in many plants, particularly monocotyledonous plants, owing to its relatively strong host specificity as well as practical problems such as tissue browning during co-cultivation [2]. Biolistic gene delivery is also suitable for the rapid delivery of large DNA fragments, but it is often associated with high cost, relatively low transformation efficiency, random genomic integration, and tissue damage [3]. In contrast, CNTs-based genetic transformation methods enable the efficient, non-toxic, and mechanical-aid-free delivery of exogenous genes, thereby overcoming the limitations of current plant transformation techniques [2]; however, their application in plant genetic delivery is still at an early stage. In addition to these major approaches, mesoporous silica- and carbon dot-mediated deliveries have also been explored in *Arabidopsis thaliana* and wheat (*Triticum aestivum*), respectively [4,5]. The former has shown promise due to its high DNA loading capacity, whereas the latter has been reported to enable gene editing. Nevertheless, for these two systems, their relative performance in terms of stable genomic integration versus transient expression remains unclear.

CNTs have successfully mediated genetic transformation in various plant species. For instance, single-walled carbon nanotubes (SWNTs) have facilitated DNA delivery in the protoplasts of tobacco (*Nicotiana tabacum*), common rue (*Ruta graveolens*), wheat, upland cotton (*Gossypium hirsutum*), and arugula (*Eruca vesicaria* subsp. *sativa*), achieving a high protein expression level without transgene integration [6]. Using carboxylated single-walled carbon nanotubes (COOH-SWNTs) as a template, Liu et al. (2024) [7] synthesized polyethyleneimine (PEI)-modified COOH-SWNT complexes (PEI-SWNTs) to deliver fluorescent plasmids (such as *RED* and *UBQ10*-*EGFP*) into oilseed rape (*Brassica napus*) protoplasts and tobacco leaves. Additionally, Jeong et al. (2026) [8] developed a novel nanocarrier based on hexahistidine peptide-functionalized SWNTs (His6-SWNTs). When the His6-SWNTs complex was used to deliver synthetic non-coding RNA (*STTM396*) into the cytoplasm, *STTM396* acted as a target mimic of *miR396*, preventing its binding to growth-regulating factors (*GRFs*). This interaction promoted bud formation and doubled the bud regeneration efficiency in both *Arabidopsis* and tomato (*Solanum lycopersicum*) callus. Furthermore, CNTs can also mediate gene silencing and facilitate CRISPR technology. For example, Demirer et al. (2020) [9] utilized CNTs to deliver siRNA to tobacco (*Nicotiana benthamiana*) cells and effectively silenced endogenous genes. By using CNTs to transport plasmids carrying CRISPR genomes, gene editing tools can express and edit plant DNA for a long time before decomposition, leaving no trace of modification. Dunbar et al. (2022) [10] reported that the CRISPR-Cas vector targeting the eight-hydrolycopene desaturase (*PDS*) gene was delivered into mature rice embryos using CNTs, which could allow for heritable gene editing.

Recently, morphogenic regulators such as BABY BOOM (BBM) and WUSCHEL (WUS) transcription factors have been increasingly utilized to enhance plant transformation efficiency. BBM, a member of the APETALA2 (AP2) family, contains an AP2/ETHYLENE RESPONSE FACTOR (ERF) domain and participates in various developmental processes, including cell proliferation, bud formation, somatic embryogenesis (SE), and induced regeneration [11,12]. For example, overexpression of *BBM* can promote SE in *Arabidopsis* seedlings [13]. WUSCHEL (WUS) is a member of the WUS-related homeobox (WOX) family, which plays a vital role in embryo development, organogenesis, morphogenesis, and cell division [14,15,16]. Previous studies have been reported that overexpression of *WUS* promotes SE in plant species such as alfalfa (*Medicago truncatula*), tulip tree (*Liriodendron chinense*), and cotton (*G. hirsutum*) [17]. Several studies indicate that both individual *BBM* and *WUS* genes and their co-transformation can improve transformation efficiency. For instance, the regeneration and transformation efficiency of transgenic apple (*Malus domestica*) was dramatically increased by overexpressing *MdBBM1* [18]. Overexpression of *WUS* in snapdragon (*Antirrhinum majus*) significantly increased transformation efficiency and produced more transgenic buds [19]. In addition, after using the *BBM*/*WUS2* and phosphomannose isomerase (PMI/mannose) selection systems in maize (*Zea mays*), callus induction (27.55%), regeneration (48.42%), and transformation (54.88%) efficiency were noticeably improved [20]. Moreover, the co-transformation of *ZmBBM*-*ZmWUS* in watermelon (*Citrullus lanatus*) achieved a transformation efficiency of 22.1% [21].

*Phyllostachys edulis*, belonging to the Gramineae family (genus *Phyllostachys*), is a sympodial bamboo species. Due to its long flowering cycle, its reproduction mainly relies on vegetative propagation. The mature embryos of *P. edulis* have been used as explants. On an MS (Murashige and Skoog) basal medium supplemented with 2,4-D and Zeatin (ZT), tightly packed, light yellow callus tissues were induced. Approximately 5% of the cells in these tissues could directly differentiate into shoots, thus establishing the regeneration system for *P. edulis* [22]. However, issues such as difficulties in explants’ disinfection and low regeneration frequency still remain. In addition, immature embryos of *P. edulis* have also been used as explants to induce callus formation and adventitious shoot differentiation. Through the *Agrobacterium tumefaciens*-mediated transformation of *P. edulis* callus, an in vitro regeneration and genetic transformation system was established, and transgenic plants were obtained [23]. Nevertheless, this generally required three to four months to obtain regenerated plants, and the transformation efficiency was only about 5%. By adopting wounding and vacuum infiltration pretreatment, agrobacterium-mediated gene delivery was achieved in *P. edulis*. This transformation system was verified to enable the successful heterologous expression of exogenous genes (such as the *RUBY* and *Cas9* genes) in *P. edulis* leaf, but the foreign DNA did not integrate into the genome [24]. Furthermore, the genetic transformation system of *P. edulis* leaves mediated by bamboo mosaic virus has been established, in which the exogenous-enhanced green fluorescent protein gene (*EGFP*) and red underlying beauty yielding (*RUBY*) reporter gene were successfully expressed [25]. These findings have provided diverse technical platforms for functional genomic studies. However, the establishment of a stable genetic transformation system for *P. edulis* still faces challenges such as a long regeneration cycle, low transformation efficiency, and poor differentiation efficiency.

To establish genetic transformation system for *P. edulis* mediated by carbon nanotube, this study used immature embryos to induce callus tissues and utilized SWNTs-assisted DNA delivery technology to transfer the *PeBBM2* and *PeWUS* genes into the callus tissues of *P. edulis*. The regulatory effects of these genes in callus and shoot formation of *P. edulis* were explored. This study will lay the foundation for the construction of a genetic transformation system of *P. edulis* using growth regulators, and provide technical support for gene function analysis and molecular breeding.

## 2. Materials and Methods

### 2.1. Callus Induction

A total of 1800 immature seeds were collected, with three embryo samples of different sizes, 600 of each size. Seeds were washed with soap for 10 min, and then rinsed under running water for 30–45 min. After transferring to the aseptic conditions in a laminar flow hood, the seeds were completely immersed in 75% (v/v) ethanol for 30 s in a flask. Subsequently, they were fully submerged in a 28.8% (v/v) sodium hypochlorite solution containing a surfactant and subjected to vacuum infiltration using a vacuum pump (20 kPa, adjusted until the vacuum gauge reading reached 0.08; Automatic Science Instrument Co., Ltd., Tianjin, China) for 15 min. The seeds were then rinsed three to five times with sterile distilled water. Seed coats were removed with forceps and scalpels, the seeds were cut open, and the embryos were carefully excised and aseptically transferred onto callus induction medium (CIM). The cultures were incubated in the dark at 25 °C in a chamber. After 30 days, we observed the growth process of the callus tissue and calculated the induction frequency. Contamination rate (%) was calculated as (no. of contaminated explants/no. of inoculated explants) × 100%, and callus induction frequency (%) as (no. of embryos forming callus/no. of inoculated embryos) × 100%. Analysis of variance was performed using SPSS 24.0 software.

The CIM was optimized based on previous research [26]. It was based on Woody Plant Medium (WPM) supplemented with 2.0 mg·L^−1^ 2,4-D, 1.0 mg·L^−1^ 1-naphthaleneacetic acid (NAA), 1.0 mg·L^−1^ 6-benzylaminopurine (6-BA), 0.5 mg·L^−1^ kinetin (KT), 0.01 mg·L^−1^ Picloram (Pic), 20 g·L^−1^ sucrose, 1.0 g·L^−1^ activated charcoal (Ac), and 3.8–4.4 g·L^−1^ Gelrite, with the pH adjusted to 5.8.

### 2.2. Hygromycin Selection

Induced callus were transferred to the callus induction medium supplemented with varying concentrations of hygromycin (Hyg): 110, 130, 150, 170, and 190 mg·L^−1^. Medium without hygromycin served as the control. After one month of dark incubation at 25 °C, callus browning was observed and recorded. Each treatment included seven callus tissue blocks, and three biological replicates were performed.

### 2.3. Vector Construction

Hydroponically, seedlings of *P. edulis* grown for approximately two months were collected from a growth chamber at Zhejiang Agriculture Forestry University. Growth conditions were as follows: 18–25 °C, 16 h light/8 h dark photoperiod and light intensity of 250–350 μmol·m^−2^·s^−1^. Total RNA was extracted from seedling shoots using the SteadyPure Plant RNA Extraction Kit (Accurate Biology, Changsha, China), and reverse transcription was performed using the Hifair^®^ III 1st Strand cDNA Synthesis SuperMix for qPCR (gDNA digester plus) (Yeasen Biology, Shanghai, China).

In this study, both the genomic sequence (*PeWUS*-DNA, containing introns) and the coding sequence (*PeWUS*-cDNA, without introns) were cloned. This dual strategy was employed because introns can often enhance mRNA accumulation through intron-mediated enhancement and help mitigate the potential post-transcriptional gene silencing risks frequently associated with the robust overexpression of developmental regulators [27,28].

Using the cDNA as a template, *PeBBM2*, *PeWUS*-DNA, and *PeWUS*-cDNA were amplified. The *PeWUS*-cDNA coding sequence was segmented into three fragments (Insert 1, Insert 2, and Insert 3) based on the exon positions used for cloning. Primer sequences are provided in Table S1. The original pMDC43 plasmid was preserved by our team, and the original P4282-RUBY plasmid was presented by Jiankang Zhu’s team. The pMDC43 plasmid was used as a template to amplify the 35S-HygR and UBQ10-eGFP fragments. The P4282-UBQ10-eGFP-35s-polyA vector was linearized using the *Xho* I restriction enzyme (with a pre-existing *Xho* I site between 35s and polyA; New England Biolabs, Ipswich, MA, USA). The *PeBBM2* fragment was inserted into the linearized vector via homologous recombination. Subsequently, the 35S-HygR fragment was inserted into the vector linearized by *Pme* I digestion, thereby obtaining the P4282-PeBBM2-eGFP-HygR vector. The P4282-PeWUSDNA-eGFP-HygR, P4282-PeWUScDNA-eGFP-HygR, p4282-PeWUSDNA-p2a-PeBBM2-eGFP-HygR, and p4282-PeWUScDNA-p2a-PeBBM2-eGFP-HygR vectors were constructed using the same methodology (primer sequences are provided in Table S1; vector construction workflows are summarized in Figure S1). The plasmid with only marker genes, *GFP* and *HygR*, was used as the control, namely p4282-eGFP-HygR (empty vector).

### 2.4. Callus Transformation Mediated by Single-Walled Carbon Nanotubes

The preparation of single-walled carbon nanotube suspension was based on a previous study [6]. Briefly, 30 mg of COOH-SWNTs powder (Sigma-Aldrich, Darmstadt, Germany) was dispersed in 30 mL sterile Milli-Q water (Merck, Darmstadt, Germany), and sonicated in a water bath at room temperature for 10 min to yield a black suspension. The suspension was then placed in an ice bath and processed using an ultrasonic homogenizer at an amplitude of 30–50 W for 30–60 min. Afterward, the mixture was left undisturbed at room temperature for 10 min. The suspension was centrifuged at 12,000 rpm for 1 h at room temperature. Subsequently, 2–3 mL supernatant was carefully collected and transferred to a new centrifuge tube. An aliquot of the prepared COOH-SWNTs suspension was diluted 10-fold, and its concentration was determined using spectrophotometer at a wavelength of 632 nm (extinction coefficient: 0.036 L·mg^−1^·cm^−1^), yielding a final concentration ranging from 100 to 150 mg·L^−1^. To activate the carboxyl groups, an appropriate volume of 500 mM MES buffer (pH 4.5–5.0) was added to the COOH-SWNTs suspension to achieve a final MES concentration of 100 mM (pH 4.5–6.0). Separately, an activating solution was prepared by dissolving 10 mg N-(3-dimethylaminopropyl)-N’-ethylcarbodiimide hydrochloride (EDC) and 10 mg N-hydroxysulfosuccinimide sodium salt (NHS) in 2.5 mL 100 mM MES buffer (pH 4.5–5.0). The EDC/NHS solution was then added dropwise to the COOH-SWNTs suspension at room temperature, followed by sonication for 15 min. The mixture was subsequently incubated on a shaker at 180 rpm for 1 h at room temperature. To remove free EDC, NHS, and unreacted by-products, the activated COOH-SWNTs suspension was purified using centrifugal filter units. Prior to use, two filter units were pre-washed with 15 mL 0.1 × PBS (pH 7.4) by centrifuging at 10,000 rpm for 2 min. The activated SWNTs solution was divided equally into the two filters, and the volume in each was adjusted to 50 mL with 0.1 × PBS. The samples were centrifuged at 300× *g* for 8 min at 21 °C. This washing procedure was repeated three times, and the retentate was briefly vortexed after each cycle. Upon completion, the filter units were sonicated in a water bath at room temperature for 1 min and briefly vortexed. The purified COOH-SWNTs from both filters were then combined into a single 50 mL centrifuge tube, diluted with 0.1 × PBS, sealed, and sonicated in a water bath for 15 min at room temperature. PEI was dissolved in 0.1 × PBS to a final volume of 5 mL, and the pH was adjusted to 7.4–7.6 using 5 M HCl. The activated COOH-SWNTs suspension was gradually added to the PEI solution, and the pH of the resulting mixture was adjusted to 7.0–8.0 using 5 M HCl or 10 N NaOH. Finally, the mixture was incubated on a shaker at 180 rpm for 16 h at room temperature to facilitate the formation of micro-aggregates.

The PEI-SWNTs and plasmid DNA were diluted to a concentration of 40 ng·μL^−1^. The transformation complex was prepared by mixing 125 μL PEI-SWNTs with 833 μL 2-Morpholinoethanesulfonic Acid (MES) delivery buffer, followed by the addition of 42 μL plasmid DNA (the six plasmids mentioned in the section of Vector Construction). The mixture was gently flicked with fingertips and pipetted repeatedly to ensure thorough mixing. The DNA-PEI-SWNTs complex was formed by incubating the mixture at room temperature for 1 h.

*P. edulis* callus samples with good growth conditions were selected and suspended for three days. They were then transferred to a sterile conical flask, and the DNA-PEI-SWNTs complex was added to completely submerge the callus. The mixture was incubated on a shaker at 28 °C and 140 rpm in the dark for 2 h. Following treatment, the callus were placed on sterile filter paper to remove excess liquid, transferred to subculture medium, and incubated in the dark at 25 °C for six days. Once the callus resumed growth, they were transferred to selection medium containing 150 mg·L^−1^ hygromycin. Selection was conducted in the dark with subculturing every four weeks; resistant callus were typically obtained after three subcultures. Finally, the selected callus were transferred to differentiation medium and incubated in a greenhouse under light conditions.

The composition of the callus subculture medium (CSM) is WPM, 2.0 mg·L^−1^ 2,4-D, 1.0 mg·L^−1^ NAA, 1.0 mg·L^−1^ 6-BA, 0.5 mg·L^−1^ KT, 1 g·L^−1^ Ac, 20 g·L^−1^ Sucrose, 3.8–4.4 g·L^−1^ Gelrite, and 0.1–4 mg·L^−1^ Imazapyr. The composition of the shoot induction medium (SIM) is MS, 0.5 mg·L^−1^ NAA, 2.0 mg·L^−1^ 6-Benzylaminopurine (BAP), 3.0 mg·L^−1^ ZT, 30 g·L^−1^ Sucrose, 0.5 mg·L^−1^ Abscisic Acid (ABA), 1 mM·L^−1^ AMP, and 3.8–4.4 g·L^−1^ Gelrite. The composition of the root induction medium (RIM) is 1/2 MS, 30 g·L^−1^ Sucrose, 2.5 g·L^−1^ Gelrite, and 2 mg·L^−1^ Indole-3-Butyric Acid (IBA). The pH values of all the above-mentioned culture media were 5.8. They were sterilized under high-pressure steam at 121 °C for 20 min. Those substances that are not heat-resistant (such as ZT) were added after the culture medium cooled to approximately 50 °C, and then the culture medium was thoroughly and evenly mixed.

### 2.5. Callus Growth Rate Analysis

Callus with uniform growth states were selected and transferred to subculture medium. Changes in callus area were monitored to calculate the growth rate before and after transformation. Photographs were taken at regular intervals under the same light source and positioning, and the callus area was measured using ImageJ software (version 2.0). Each group included four replicates. The growth increment of the callus tissue was calculated by subtracting the area of the tissue after cultivation from that before cultivation. Statistical significance was analyzed using SPSS 24.0 software.

### 2.6. Identification of Overexpressing Callus and Plantlet

Total DNA was extracted from the *PeBBM2*-overexpressing callus using a plant DNA kit (SIMGEN, Hangzhou, China) using DNA as a template to identify positive callus tissue, and the specific primers for PCR amplification are provided in Table S2.

Using the polysaccharide polyphenol RNA extraction kit (Monad Biotechnology, Suzhou, China), RNA was extracted from both the plantlets overexpressing *PeBBM2* and the seedlings induced from normal mature embryos. Reverse transcription was performed using the Hifair^®^ III 1st Strand cDNA Synthesis SuperMix for qPCR (gDNA digester plus) (Yeasen Biotechnology, Shanghai, China). RT-PCR was conducted using the specific primers of the *HygR* gene to identify positive plantlets. The specific primers are provided in Table S2.

### 2.7. Gene Expression Analysis

Based on previous study [12], six downstream genes of *BBM* were selected for relative expression analysis: Agamous-Like 15 (*AGL15*), Leafy cotyledon 1 (*LEC1*), Leafy cotyledon 2 (*LEC2*), indole-3-acetic acid 30 (*IAA30*), YUCCA (*YUC*), and Trp aminotransferase of arabidopsis 1 (*TAA1*). The RNA and cDNA mentioned above were used as the sample. Quantitative real-time PCR (qRT-PCR) was performed using Hieff^®^ qPCR SYBR^®^ Green Master Mix (Yeasen Biotechnology, Shanghai, China) following the manufacturer’s instructions. The *PeNTB* was used as a reference gene. The reaction conditions were: 95 °C for 30 s; followed by 40 cycles of 9 °C for 10 s and 60 °C for 30 s; and a final storage at 12 °C. Data were analyzed using the 2^−ΔΔCT^ method. Primer sequences are listed in Table S3. The analysis of differences in relative expression levels was conducted using the independent sample *t*-test in SPSS 24.0 software. * *p* < 0.05 and ** *p* < 0.01. A schematic diagram of the experimental workflow is shown in Figure S2.

## 3. Results

### 3.1. Induction of P. edulis Callus

**Figure 1:**
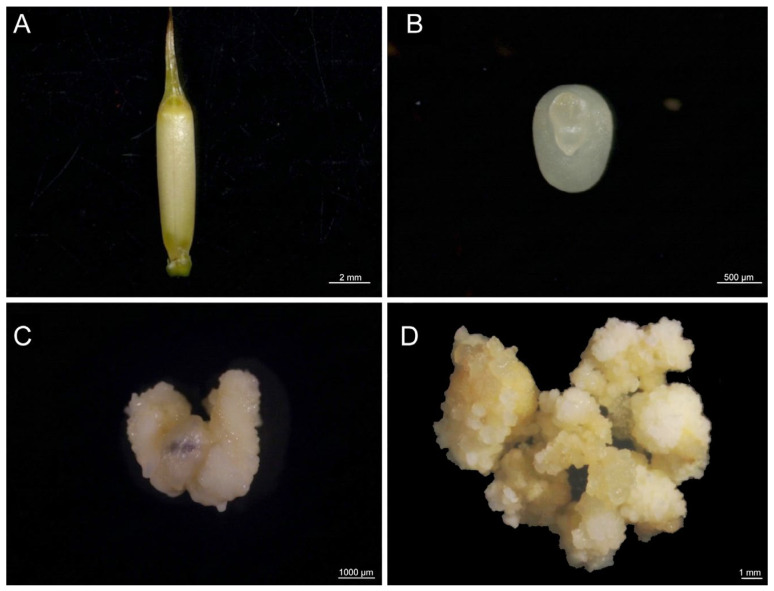
Process of callus induction of *P. edulis*. (**A**) Immature seeds of *P. edulis*. (**B**) Immature zygotic embryo. (**C**) Callus induced from immature zygotic embryos. (**D**) Proliferation of induced callus. The white lines are scale bars.

Embryos of different sizes were inoculated onto the optimized callus induction medium. Under the condition of a subculture cycle of 15 to 30 days, callus tissue was successfully induced (Figure 1). As shown in Table 1, the size of embryos significantly influenced the callus induction rate. The 0.9–1.0 mm (long axis) embryos exhibited the highest callus induction rate (84.83%) and the lowest contamination rate (26.83%). In contrast, the 1.1–1.4 mm embryos showed a reduced induction rate (81.17%) and an increased contamination rate (30.33%), while the 1.5–1.6 mm embryos showed the lowest induction rate (77.17%) and highest contamination (33.17%).

### 3.2. Hygromycin Sensitivity Analysis

**Figure 2:**
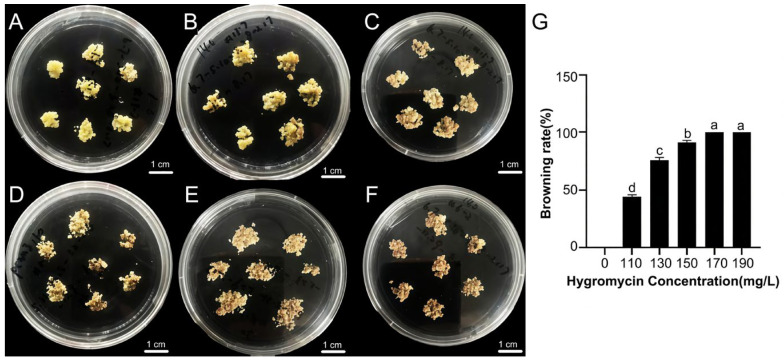
Hygromycin sensitivity of *P. edulis* callus. Growth of *P. edulis* callus after 30 days treatment with (**A**) 0, (**B**) 110, (**C**) 130, (**D**) 150, (**E**) 170, and (**F**) 190 mg·L^−1^ Hyg. Scale bar = 1 cm. (**G**) Browning rate of callus under different hygromycin concentrations. Data were analyzed by one-way ANOVA with SPSS software. Different letters represent significant differences (*p* < 0.05).

To determine the optimal hygromycin selection pressure, callus were cultured on medium containing 110, 130, 150, 170, and 190 mg·L^−1^ hygromycin, with hygromycin-free medium as the control. After one month of dark incubation at 25 °C, callus browning was observed (Figure 2A–F). At 110 mg·L^−1^, the callus showed slight proliferation. At 150 mg·L^−1^, the callus were still able to proliferate, but with obvious browning. At 170 mg·L^−1^ and 190 mg·L^−1^, more than 90% of the callus had browned (Figure 2G). Given that excessively high concentrations may lead to extensive necrosis, whereas low concentrations may increase the risk of false-positive selection, 150 mg·L^−1^ was selected as the optimal concentration for screening resistant callus.

### 3.3. Carbon Nanotube-Mediated Transformation of PeBBM2 and PeWUS in Callus

**Figure 3:**
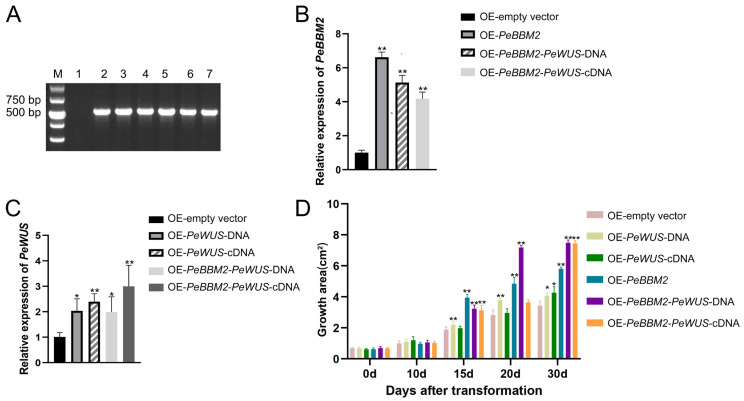
Relative expression levels of *PeBBM2* and *PeWUS* and proliferation area statistics of transgenic callus. (**A**) PCR identification of transgenic callus using the *HygR* gene. Lane M corresponds to the DNA marker. Lanes 1–7 correspond to the wild-type control (did not make any transformation), eGFP-HygR (empty vector), PeBBM2-eGFP-HygR, PeWUScDNA-eGFP-HygR, eGFP-PeWUSDNA-HygR, PeWUScDNA-PeBBM2-eGFP-HygR, and PeWUSDNA-PeBBM2-RUBY-HygR transformants, respectively. (**B**) Relative expression level of *PeBBM2* in transgenic callus. (**C**) Relative expression level of *PeWUS* in transgenic callus. (**D**) Statistical analysis of transgenic callus area. ** and * indicate significant differences at *p* < 0.01 and *p* < 0.05, respectively.

The six vectors were respectively transformed into callus using the SWNTs-mediated transformation system (Figure S3). Among them, the p4282-eGFP-HygR (empty vector) transformation callus was used as the control. Successful overexpression of *PeBBM2* and *PeWUS* genes was confirmed by RT-PCR (Figure 3A). Compared with the control, the results of qRT-PCR showed that the expression level of *PeBBM2* significantly increased in the *PeBBM2*, *PeBBM2*-*PeWUS*-DNA, and *PeBBM2*-*PeWUS*-cDNA lines (Figure 3B), and the expression level of *PeWUS* also considerably rose in the *PeWUS*-DNA, *PeWUS*-cDNA, *PeBBM2*-*PeWUS*-DNA, and *PeBBM2*-*PeWUS*-cDNA lines (Figure 3C).

The callus proliferation area was then monitored over a 30-day period (Figure 3D). During the first 10 days after transformation, no significant differences in area were observed between the gene-transformed callus and the control. From day 10 to 15, callus transformed with *PeWUS*-DNA, *PeBBM2*, *PeBBM2*-*PeWUS*-DNA, and *PeBBM2*-*PeWUS*-cDNA all exhibited significantly increased callus area compared with the control, with the *PeBBM2*-transformed callus showing the fastest increase in area at this stage. From days 15 and 20, the promotive effects became more obvious in the *PeWUS*-DNA, *PeBBM2*, and *PeBBM2*-*PeWUS*-DNA lines, among which the *PeBBM2*-*PeWUS*-DNA-transformed callus showed the biggest proliferation area, reaching almost twofold that of the control. From day 20 to 30, area remained significantly raised in the *PeWUS*-DNA, *PeBBM2*, *PeBBM2*-*PeWUS*-DNA, and *PeBBM2*-*PeWUS*-cDNA lines, with the two co-transformed groups displaying the most substantial increases. These results indicated that *PeBBM2* alone might exert a promotive effect during the early stage of callus proliferation, whereas co-expression with *PeWUS*, particularly *PeWUS*-DNA, may lead to a lasting stimulating effect in the subsequent stages.

### 3.4. Shoot Formation of PeBBM2-Overexpressing Transgenic Callus

**Figure 4:**
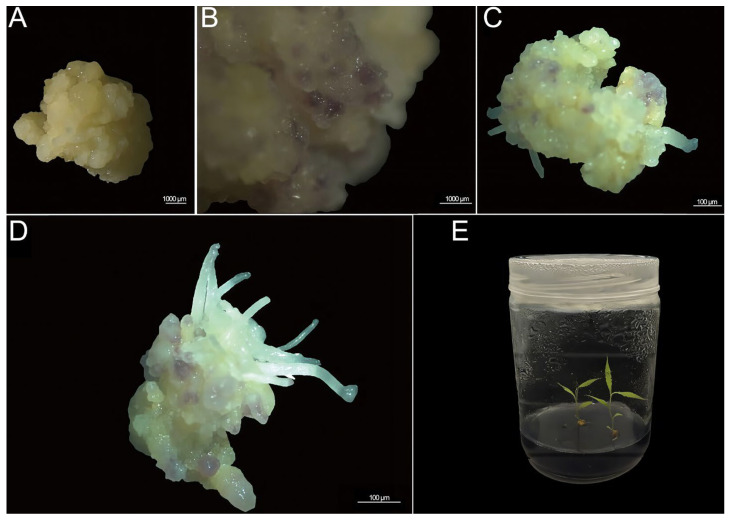
Transformation of *P. edulis* callus with the p4282-PeBBM2-eGFP vector. (**A**) The callus before transformation. (**B**) The purplish-red callus after transformation. Root differentiation of callus at (**C**) 30 and (**D**) 40 days after transformation. (**E**) *P. edulis* plantlet about 60 days after transformation. The white lines are scale bars.

After continuous culture of the transgenic callus, only callus overexpressing *PeBBM2* formed shoots and regrew into plantlets. Morphological observation showed that root initiated around 30 days after transferring to differentiation medium and was accompanied by purple coloration in the callus. The rooted callus were then transferred to the shoot induction medium under light. Shoot primordia appeared at around day 40, visible shoots emerged at around day 50, and the plantlets were obtained by day 60 (Figure 4). During the cultivation process, each callus tissue block produces five to six shoots, among which one to two shoots can grow into a complete plantlet.

In order to initially explore the molecular mechanism of the shoot formation process, the expression levels of several morphogenetic regulatory factors were analyzed in *PeBBM2*-overexpressing lines. Compared with the wild type, the expression levels of *IAA30* and *YUC* were significantly upregulated in overexpressing lines, whereas *AGL15* was significantly downregulated. In contrast, the expression levels of *TAA1*, *LEC1*, and *LEC2* showed no obvious changes (Figure S4). These results suggest that *PeBBM2* may promote callus and shoot formation, at least in part, by upregulating genes such as *IAA30* and *YUC*.

## 4. Discussion

As a representative bamboo species in China, with the widest distribution and high economic and ecological value, *P. edulis* exhibits a unique flowering habit that makes sexual hybrid breeding extremely challenging, rendering molecular breeding a key pathway for germplasm innovation. At present, the genome of *P. edulis* has been sequenced and numerous candidate functional genes have been identified; however, the lack of a stable and efficient genetic transformation system has severely constrained molecular biological studies and genetic improvement in this species. Morphogenetic regulators can markedly enhance plant regeneration and transformation efficiency and are important for establishing an efficient genetic transformation system. In this study, we introduced *PeBBM2* and *PeWUS* into *P. edulis* callus using an SWNTs-mediated method and explored their potential regulatory effects on the callus and shoot formation. This work provides technical support for subsequent functional gene identification, function analysis, and molecular breeding in *P. edulis*.

The induction of callus from immature embryos is a key step in plant tissue culture and genetic transformation and is widely applied in the establishment of regeneration systems in gramineous crops such as wheat, maize, and rice [29,30,31]. A variety of explant types have been used for the tissue culture of *P. edulis*, including mature zygotic embryos, shoot tips, and young stems and leaves [32,33,34]. In this study, immature zygotic embryos of *P. edulis* were selected as explants and successfully induced to form callus (Figure 1). Previous studies have shown that 2,4-D is crucial for callus induction in *P. edulis*. Using a medium composed of MS + 4 g·L^−1^ 2,4-D + 30 g·L^−1^ sucrose + 8 mg·L^−1^ agar at pH 5.8, the callus induction efficiency can reach approximately 34% [26]. Based on this formulation, we further modified the medium by adding plant growth regulators such as NAA and 6-BA and achieved an induction efficiency of about 85% at pH 5.8 (Table 1). In addition, through gradient tests, we confirmed that a medium containing 150 mg·L^−1^ hygromycin was optimal for the subsequent selection of positive callus.

To overcome genotype-dependent differences in regeneration capacity, many studies have enhanced the transformation efficiency and regeneration ability of plants by overexpressing morphogenetic regulators [17,18,21,35,36]. In monocotyledonous plants, overexpression of *BBM* and *WUS* genes can promote SE or shoot regeneration. In maize, the co-transformation of *ZmBBM* and *GRF4*-*GIF1* genes promotes SE and significantly enhances the gene transformation efficiency [37]. Moreover, after overexpressing *BBM*-*WUS* in wheat, transformation efficiency can be increased by five to six times, and transgenic plants can be obtained from immature embryos within just 60 days [38]. As a monocotyledonous plant, overexpression of the above two genes in *P. edulis* may also produce similar effects. However, no relevant reports have been disclosed to date. In this study, we transformed individually and co-transformed *PeBBM2* and *PeWUS* into *P. edulis* callus. Phenotypic observations showed that callus co-transformed with *PeBBM2*-*PeWUS* exhibited the largest proliferation areas, and the callus areas at 30 days in the *PeBBM2* and *PeBBM2*-*PeWUS* treatments were more than threefold that at day 0 (Figure 3). These results indicate that the overexpression of *PeBBM2* and *PeWUS* in the *P. edulis* callus tissue may promote its proliferation.

Previous studies have demonstrated that CNTs can passively traverse the cell wall and plasma membrane of callus cells, thereby enabling the intracellular delivery of DNA into callus cells or subcellular compartments such as mitochondria [10,39,40,41]. Moreover, CNTs can protect loaded nucleic acids from intracellular nuclease degradation [42], which helps maintain exogenous DNA integrity and supports subsequent transcription and expression. Surface modification of CNTs, for example with PEI or chitosan, can further enhance the efficiency and specificity of gene delivery. PEI-functionalized CNTs carry positive charges and can therefore form stable complexes with negatively charged DNA through electrostatic interactions. These complexes may also interact more effectively with the negatively charged plant cell membrane, thereby improving cellular uptake [6]. Callus tissue consists of undifferentiated cells cultured in vitro and is characterized by active cell proliferation, a relatively loose cell wall structure, open chromatin status, and frequent DNA replication and repair activities. Compared with mature plant cells, these features may increase the likelihood that the exogenous DNA introduced into callus cells is retained and stably maintained. At present, the transformation of *P. edulis* is mainly mediated by *A. tumefaciens*. In the present study, by using SWNTs as a medium, we achieved the delivery and expression of *PeBBM2* and *PeWUS* genes in the *P. edulis* callus (Figure 4). We obtained plantlets overexpressing *PeBBM2* gene; this might be caused by the partial integration of exogenous DNA. However, the possibility that *PeBBM2* was merely transiently expressed cannot be excluded. Further molecular evidence is needed to determine this.

*BBM* is widely recognized as an important regulator of plant cell totipotency and has been reported to influence the expression of multiple transcription factors involved in growth and development. In Arabidopsis, chromatin-based analyses in somatic embryo-related tissues have suggested that *BBM* can associate with the promoter region of *AGL15*, implicating *AGL15* as a component of the *BBM*-associated embryogenic regulatory network [43,44]. In the present study, qRT-PCR analysis was performed using *P. edulis* plantlets leaves derived from *PeBBM2*-overexpressing callus, with leaves from plantlets induced by mature embryos used as the control. Under these conditions, *AGL15* expression was significantly downregulated in the *PeBBM2*-overexpressing plantlets (Figure S4). This pattern may be consistent with the possibility that the relationship between *BBM* and *AGL15* is stage-dependent, as previous studies in Arabidopsis have suggested that the *BBM*-associated activation of *AGL15* may occur mainly during the induction or early stages of SE, whereas its expression may decline after regeneration proceeds beyond the embryogenic phase [43,44,45]. Previous studies have also linked *BBM* to auxin-related pathways, including regulation of the *YUC* gene involved in auxin biosynthesis [17,24]. Because *YUC*-mediated auxin production is important for regeneration-related processes such as de novo root organogenesis and shoot regeneration [46,47], elevated *YUC* expression is observed in *PeBBM2*-overexpressing plantlets. We speculate that *PeBBM2* overexpression is associated with altered auxin-related developmental regulation in the tissues after shoot formation. Likewise, *IAA30*, an auxin-responsive component reported to be associated with SE efficiency [48,49], was also upregulated in the *PeBBM2*-overexpressing plantlets. Rather than indicating a simple positive or negative regulatory effect, *IAA30* might participate in fine-tuning auxin-response outputs within the developmental context associated with *PeBBM2* overexpression [50]. We suppose that its upregulation may therefore reflect a reshaping of the auxin-response network in the plantlets. However, since these expression analyses were conducted on the leaf tissues of *PeBBM2*-overexpressing plantlets rather than the materials from the early induction stage of callus, we cannot clarify the regulatory relationships during the SE or callus induction process at present. Further stage-specific analysis is needed to determine whether *PeBBM2* directly or indirectly affects these downstream genes to promote the regeneration of *P. edulis*.

## 5. Conclusions

In this study, we established a callus induction system for *P. edulis* using immature embryos and identified 150 mg·L^−1^ as the optimal Hyg concentration for transformation selection. We successfully applied SWNTs-mediated DNA delivery to transform *PeBBM2* and *PeWUS* into *P. edulis* callus. Co-transformation of *PeBBM2* and *PeWUS* significantly increased callus formation. Furthermore, expression analysis indicated that *PeBBM2* overexpression was associated with increased expression of *IAA30* and *YUC* and decreased expression of *AGL15*, providing insight into the regulatory mechanism in *P. edulis*. Taken together, these results lay the foundation for developing a genetic transformation system for plants using carbon nanotube-mediated DNA delivery and morphogenetic regulators.

## Figures and Tables

**Table 1:** Induction efficiency of immature embryo callus in *P. edulis*.

The Size of Immature Embryo (Long Axis, Unit: mm)	Rate of Contamination	Induction Efficiency
0.9–1.0	26.83 ± 1.72 c	84.83 ± 2.48 c
1.1–1.4	30.33 ± 2.34 b	81.17 ± 1.17 b
1.5–1.6	33.17 ± 2.14 a	77.17 ± 2.04 a

Data are presented as mean ± standard error. Different letters within the same column indicate significant differences at *p* < 0.05. Analysis of variance was performed using SPSS 24.0 software.

## Data Availability

The data obtained in the experiment are presented in the manuscript and the Supplementary Materials.

## Supplementary Materials

The following supporting information can be downloaded at: https://www.mdpi.com/article/10.3390/genes17060598/s1, Figure S1: Flow chart of vector construction. (A) p4282-eGFP-HygR vector. (B) p4282-PeBBM2-eGFP-HygR vector. (C) p4282-PeWUSDNA-eGFP-HygR vector. (D) p4282-eGFP-PeWUScDNA-HygR vector. (E) p4282-PeBBM2-p2a-PeWUSDNA-eGFP-HygR vector. (F) p4282-PeBBM2-p2a-PeWUScDNA-eGFP-HygR vector; Figure S2: A schematic diagram of the experimental workflow; Figure S3: Phenotypes of transgenic callus. (A) The transgenic callus tissue after Hyg screening. (B) Callus proliferation stage. (C) Callus differentiation stage; Figure S4: Expression patterns of several morphogenetic regulatory factor genes in wild-type and transgenic plantlet of *PeBBM2*. * *p* < 0.05 and ** *p* < 0.01; Table S1: The primers used for vector construction in *P. edulis*; Table S2: The primers used for identification of transgenic callus and plantlet; Table S3: The primers used for qRT-PCR in *P. edulis*.
