# Supplementary figures and images for: Overexpression of PeBBM2 and PeWUS Genes via Carbon Nanotube-Based DNA Delivery Enhances the Callus and Shoot Formation in Phyllostachys edulis
Source: Genes (Basel). 2026 May 22;17(6):598. doi: 10.3390/genes17060598 (PMC13299642; doi:10.3390/genes17060598)

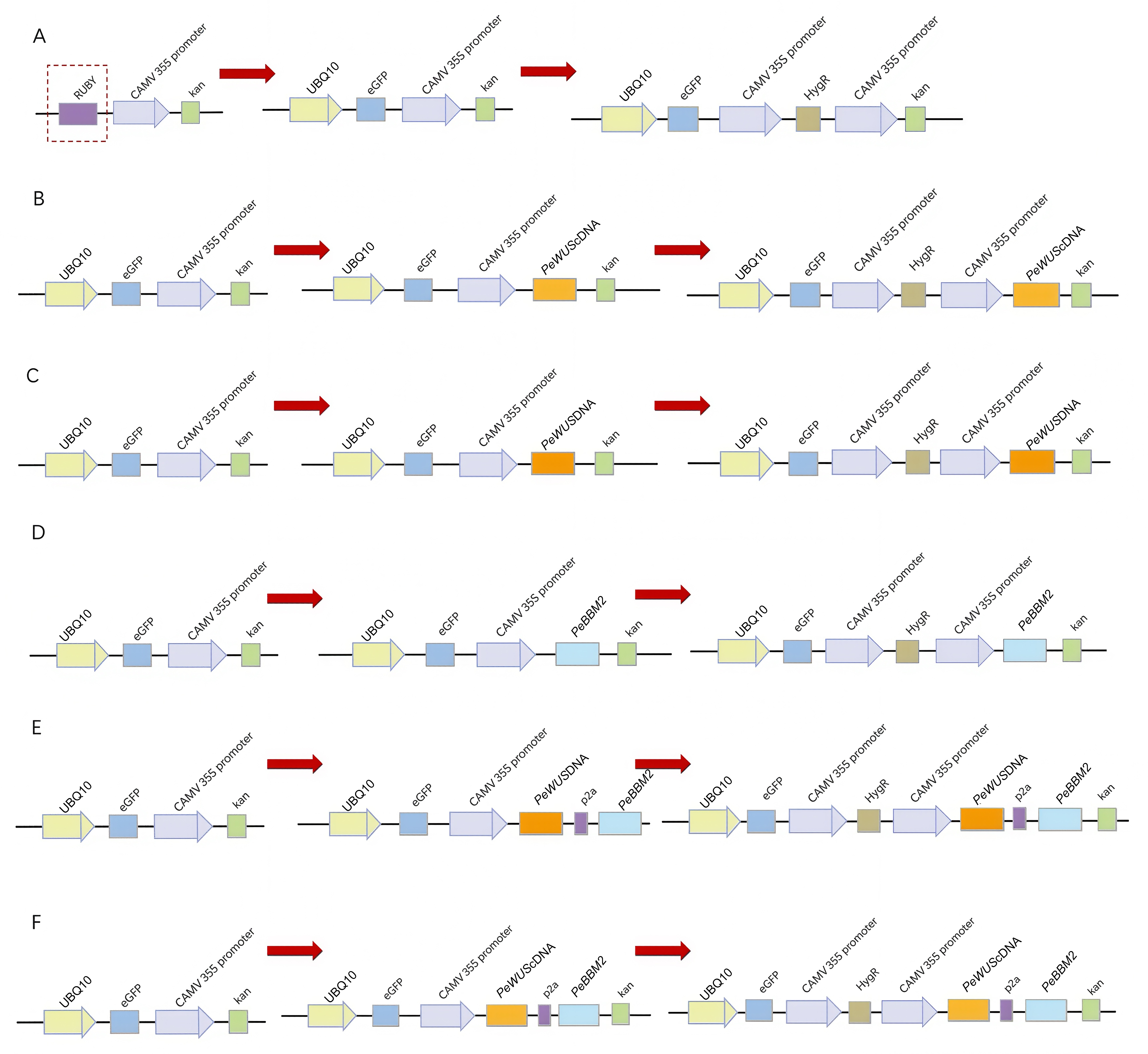

Supplement: Supplementary file 1 [file genes-17-00598-s001.zip › genes-4303404-supplementary/Supplement/Figure S1.tif]

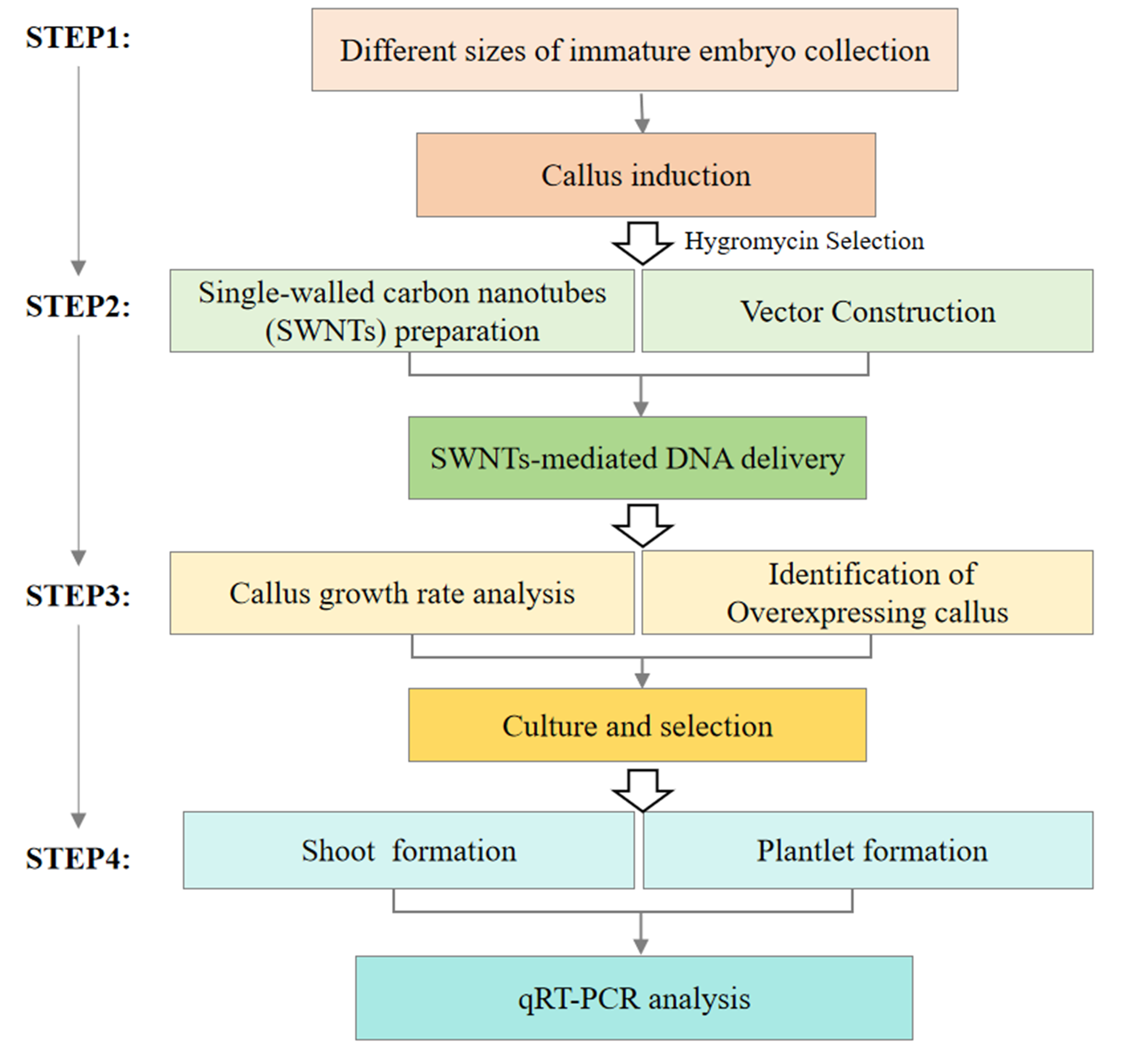

Supplement: Supplementary file 1 [file genes-17-00598-s001.zip › genes-4303404-supplementary/Supplement/Figure S2.tif]

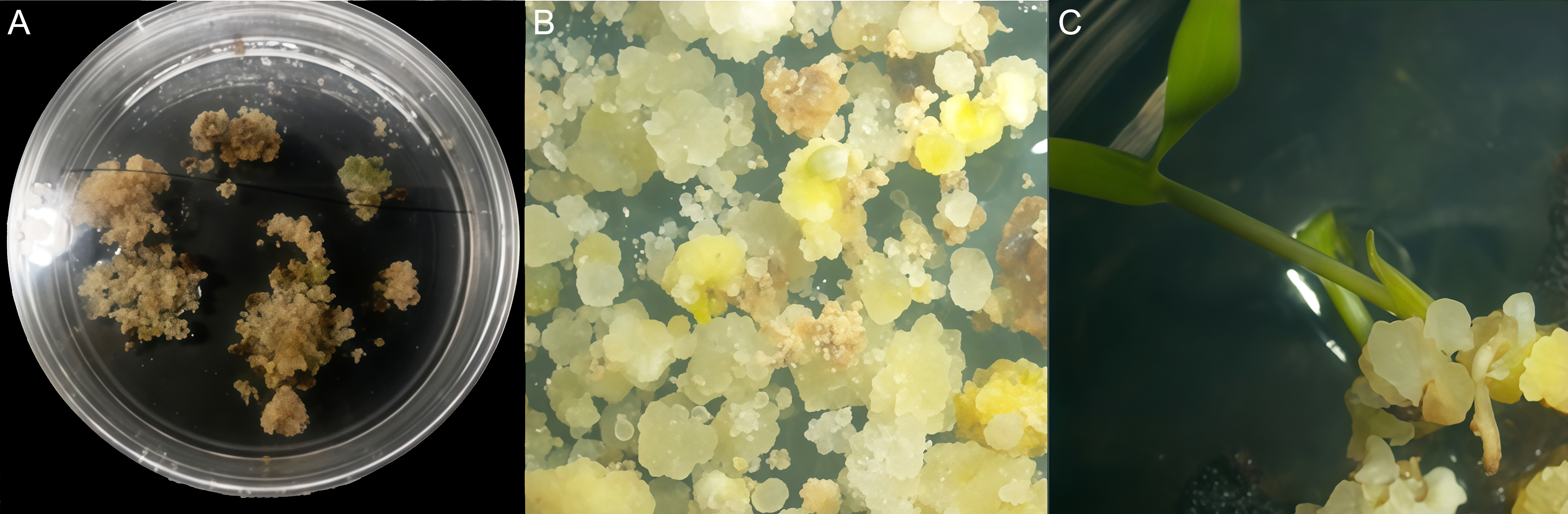

Supplement: Supplementary file 1 [file genes-17-00598-s001.zip › genes-4303404-supplementary/Supplement/Figure S3.tif]

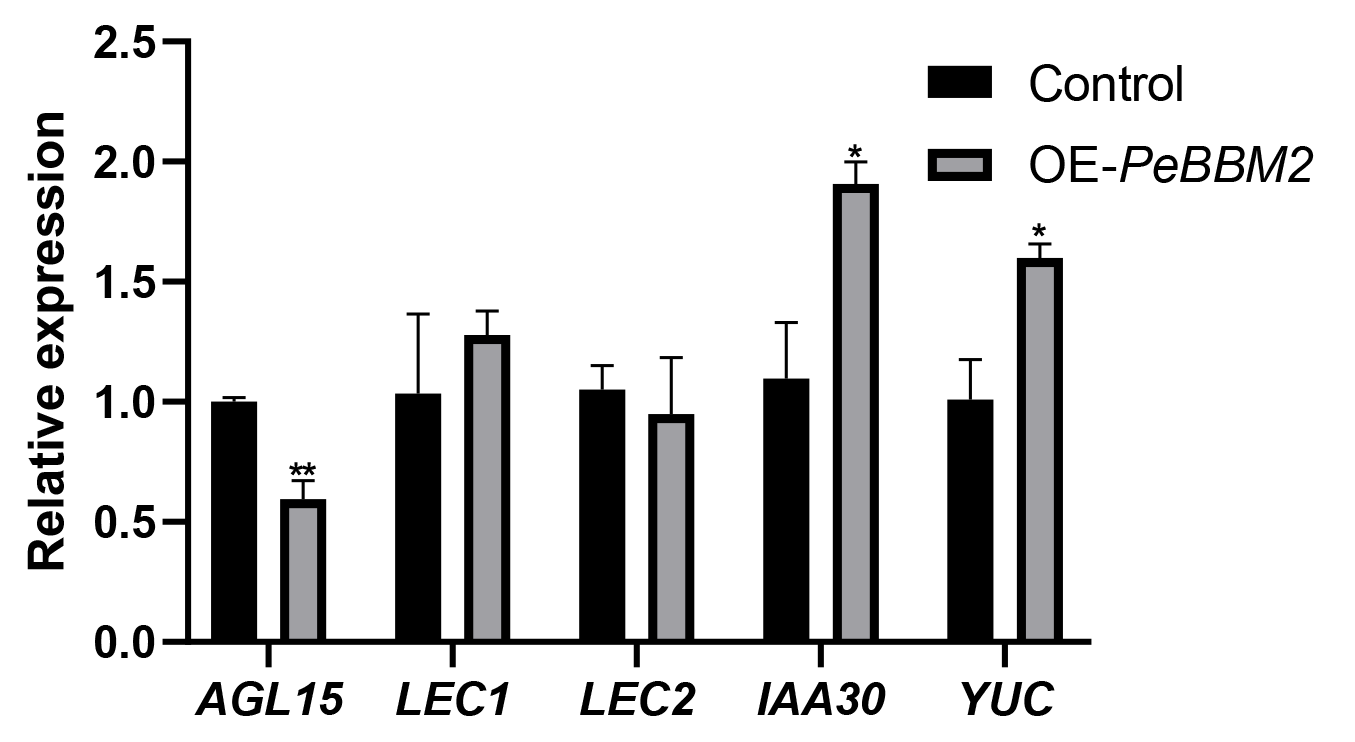

Supplement: Supplementary file 1 [file genes-17-00598-s001.zip › genes-4303404-supplementary/Supplement/Figure S4.tif]
